# Does generative AI mean the “end of history” for pharmacovigilance automation? towards a framework for the future of human-AI systems

**DOI:** 10.3389/fdsfr.2026.1846339

**Published:** 2026-08-18

**Authors:** Leihong Wu, Joshua Xu, Oanh Dang, Robert Ball

**Affiliations:** 1 Division of Bioinformatics and Biostatistics, National Center for Toxicological Research, Food and Drug Administration, Jefferson, AR, United States; 2 Offices of Surveillance and Epidemiology, Center for Drug Evaluation and Research, Food and Drug Administration, Silver Spring, MD, United States

**Keywords:** AI, computability, human-AI systems, large langauge models, pharmacovigilance (MeSH)

## Abstract

**Introduction:**

Advances in generative artificial intelligence (AI), particularly large language models (LLMs), have sparked discussions in automating pharmacovigilance (PV) workflows. It remains unclear whether these technological advancements fundamentally change the prior conclusions that full automation of Individual Case Safety Report (ICSR) processing is not feasible.

**Methods:**

This perspective examines recent developments in AI for PV and introduces a conceptual framework of “computable PV,” in which tasks are evaluated based on their computational tractability and suitability for automation.

**Results:**

Routine, well-defined PV tasks, including completeness checks, detection of duplicated ICSRs, and structured information extraction, are increasingly amenable to automation. In contrast, complex activities such as case-level causality assessment remain difficult to formalize and continue to rely on expert judgment. The emergence of LLMs enables broader, cross-task capabilities compared to traditional task-specific, “small” models, but introduces challenges related to reliability, auditability, and governance. As a result, hybrid architecture combining large models, small models, and rule-based components is increasingly necessary.

**Conclusion:**

Generative AI, as of today, does not signal full automation of PV but rather shifts toward hybrid human-AI systems. While AI can augment efficiency and support evidence synthesis, final decisions must remain under human oversight. Future PV systems should prioritize transparency, validation, and the integration of AI outputs into expert-driven decision-making.

## Introduction

The US FDA defines pharmacovigilance (PV) as “all scientific and data gathering activities relating to the detection, assessment, and understanding of adverse events” (FDA, 2005). Much of the work revolves around the evaluation of individual case safety reports (ICSRs) from healthcare professionals, patients, and pharmaceutical companies. These reports consist of both structured fields such as age, sex, suspect drug, and outcome and extensive free-text narratives. They are submitted in large volumes, exhibit variable quality, and require reliable processing and evaluation to meet regulatory requirements (Potter et al., 2025).

Over the last few decades, the number and complexity of ICSRs have increased steadily. PV reviewers must verify basic completeness, detect duplicate reports, determine report priority, and integrate information across multiple cases and external data sources to identify and assess potential safety signals (FDA, 2024). Each of these steps is labor intensive. Exclusive reliance on manual review is increasingly challenging, and associated delays or errors can compromise patient safety and regulatory compliance. It is therefore natural that PV has increasingly turned to artificial intelligence (AI) and machine learning (ML) for support.

In 2022, *Ball and Dal Pan* published a paper asking whether AI for pharmacovigilance, focused on the processing and evaluation of ICSRs, was “ready for prime time” (Ball and Dal Pan, 2022). That paper reviewed ML and natural language processing (NLP) methods for tasks such as case classification, seriousness, and case usefulness, mainly in the FDA Adverse Event Reporting System (FAERS, now part of the Adverse Event Monitoring System) and similar systems. Prior to the emergence of large language models (LLMs), the authors concluded that AI/ML could support several parts of the workflow, but that full automation was not realistic. Human review and judgment were still essential. The authors also pointed to gaps, including the lack of large and shared annotated ICSR datasets. Additionally, there was a lack of a “pharmacovigilance cognitive framework” defined as the “*rules of inference applied to fit-for-purpose data in ICSRs by safety reviewers to assess whether there is likely to be a causal relationship between a drug and an adverse event (AE)*” (Ball et al., 2024). More simply, the steps in case assessment were not described in a manner that could be followed by a computer, and there was no clearly defined and transparent framework for effectively integrating people, processes, and AI tools.

This lack of a “computable” description of the human PV expert’s cognitive framework remains valid today. However, the environment has changed dramatically with the emergence of foundation models that are transformer-based LLMs trained on massive datasets to understand and generate human language. These models transformed the field of NLP by demonstrating advanced capabilities on unstructured text. They can summarize narratives, extract key information, rewrite text into standard formats, and answer complex questions in natural language. This has opened new possibilities for AI in text-heavy tasks within PV, including ICSR intake, coding, deduplication, and case-series review for causality assessment (Painter et al., 2024). In parallel, regulators and other bodies have begun to issue high-level principles for applying AI and ML in medical product development and regulation (FDA, 2025; CIOMS, 2025; FDA and EMA, 2026).

This rapid advancement in AI capabilities raises a provocative question that we explore in this perspective paper: are we approaching the “end of history” for traditional safety surveillance? We are inspired to ask this question by the debate about whether AI is either an existential threat to humanity or the beginning of the golden age of human flourishing. We realized a similar debate had played out at the end of the Cold War, when Fukuyama famously invoked Hegel’s concept of history being a dialectical process to argue that the world had reached the end of history (Fukuyama, 1989).

**FIGURE 1:**
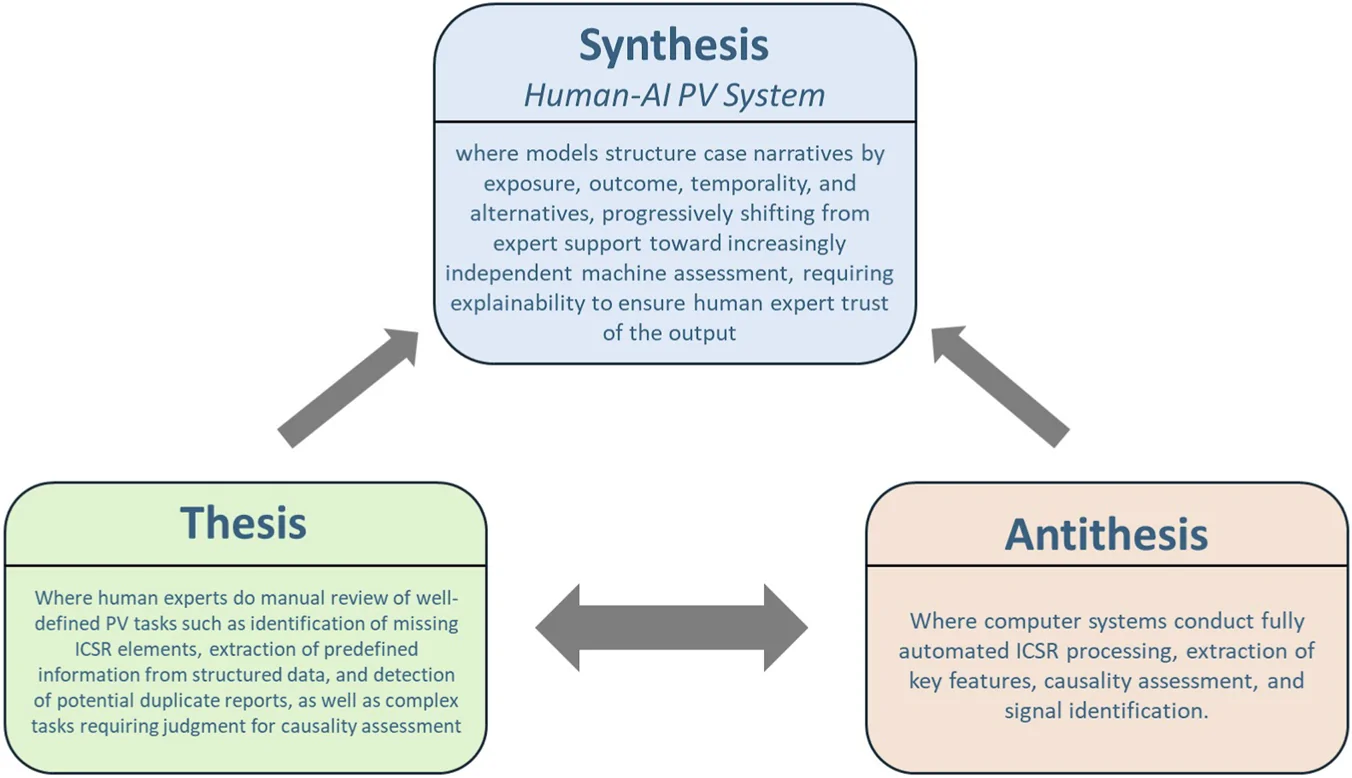
The “End of History of Pharmacovigilance (PV) automation. The dialectical process between traditional human-expert manual pharmacovigilance (PV) and fully automated Individual Case Safety Report (ICSR) processing and signal identification lead to a Human-AI PV system, where models handle most tasks but provide assessments of the decision-making process that allow humans to interpret, agree, disagree with the decisions to ensure human trust of the model output.

We extend the analogy to sharpen the focus between traditional PV and fully AI-enabled PV and point to the emergence of a practical Human-AI solution as the likely result, using the simplified expression of Hegel’s dialectical process as starting with an idea (thesis) and its opposite (antithesis) resolving through unification to a synthesis (Maybee, 2020). In Figure 1 we frame PV’s historical thesis as human expert-only, introspective judgment when applied to narrative cases, its contemporary AI-based antithesis as the claim that PV should be fully computable, independent of human experts, and its emerging synthesis as Human AI-PV systems.

Case narratives are structured by models into exposure, outcome, temporality, and alternative explanations, progressively shifting from expert support toward increasingly independent machine assessment, requiring interpretability to ensure human expert trust of the output. First, we must translate the definition of a human PV expert’s cognitive framework from the “rules of inference applied to fit-for-purpose data in ICSRs by safety reviewers to assess whether there is likely to be a causal relationship between a drug and an adverse event” (Ball et al., 2024) into a set of computable steps. By computable we refer to tasks that can be translated into sufficiently explicit and auditable computational steps, where for each step, inputs and outputs can be clearly defined, processing details can be specified with sufficient precision to enable traceable computational execution, and output quality can be evaluated using reliable and reproducible criteria. Related PV AI frameworks similarly emphasize making PV knowledge explicit and defining acceptability or quality control criteria for AI outputs in regulated environments (Mockute et al., 2019). Several PV tasks meet these conditions, including identification of missing minimum ICSR elements (FDA US, 2022), extraction of predefined information from structured data, and detection of potential duplicate reports, which involve well-defined requirements and fixed parameters, have been highlighted as particularly amenable to rule-based automation (Kassekert et al., 2022; Norén et al., 2007). In contrast, other activities, most notably full case-level causality assessment, are substantially more difficult to formalize and continue to depend heavily on expert judgment, with multiple authors noting that parts of the causal assessment reasoning process are not yet fully computable (Ball and Dal Pan, 2022; Ball et al., 2024). In practice, computability exists on a spectrum and depends on factors such as the degree of input standardization, output verifiability, tolerance for error, auditability, reliance on clinical judgment, and the regulatory consequences of false positive or false negative outcomes.

In the following sections of this paper, we will first discuss the roles of “big” models (large, general-purpose language models) and “small” models (task-specific models) in the context of PV. Then, we briefly examine the computability of some common tasks in PV, based on our past experiences and lessons learned. Finally, we outline a pragmatic, human-centered view of “computable PV” that we hope will guide realistic use of AI in this area.

## Big or small AI models?

Operationalizing the idea of computable PV requires deciding not only which workflow steps can be formalized, but also what kind of model architecture is best suited to each step. Historically, PV automation has been implemented as modular pipelines of narrowly scoped components such as NLP rule-based checks and supervised ML models for extraction, coding, and deduplication with each mapped to a specific step in ICSR processing and validated against task-specific ground truth (Ghosh et al., 2020; Kompa et al., 2022). The rise of foundation models, and especially large language models (LLMs), changes this design space in which a single pre-trained model can be adapted via prompting or light fine-tuning to perform multiple downstream tasks, including summarization and structured extraction directly from long narratives (Wornow et al., 2023). Early PV demonstrations such as using LLMs to translate natural-language reviewer questions into executable database queries illustrate how “big models” can begin to span multiple stages of the PV cognitive workflow rather than a single micro-task (Painter et al., 2024). For a perspective on computable PV, the “big versus small” distinction therefore matters because it determines whether computability is achieved through many traceable task-specific modules or through fewer general-purpose components whose flexibility must be balanced against auditability, validation burden, and governance.

This choice is already visible in PV deployments where many solutions still rely on task-specific (“small”) models and rules aligned to ICSR processing steps (Bate and Michael Tregunno, 2026), while foundation-model approaches increasingly aim to cover multiple steps using a single general-purpose (“big”) model. Here, “big” refers to LLMs/foundation models trained at scale (often multimodal) that can be repurposed across tasks, whereas “small” refers to models built or fine-tuned for one specific job (e.g., NER, deduplication) with constrained inputs and well-defined outputs.

Despite their differences in size and scope, both groups of models share a basic attribute in that any AI system used in PV needs clearly defined tasks including predictive classification and named entity recognition. Although big models can be applied to multiple tasks, the performance on each specific task is still individually evaluated. That said, both big and small models need to be evaluated with appropriate metrics and monitored for any changes in performance over time, particularly for pharmacovigilance in which safety data continually evolves. Either models require explicit decisions about where human review is integrated in the workflow and how disagreements between the model and human assessors are handled. In this sense, large language models do not remove the need for problem definition, data standards, performance benchmarks, or governance; they instead provide another approach.

Since the release of frontier LLMs, studies in medicine have shown strong performance on core NLP tasks, particularly summarization of long-form clinical text, evaluated using both automated NLP performance metrics [e.g., ROUGE (Lin, 2004), BLEU (Papineni et al., 2002), Bertscore (Zhang et al., 2019)] and clinician review (Van Veen et al., 2024; Tang et al., 2023). In PV, this matters because some PV tasks now can directly summarize case reviews without necessarily requiring model training for each narrowly defined use case (Van Veen et al., 2024). At the same time, expanding LLM use beyond summarization to extraction-style tasks (e.g., NER/concept extraction) remains an active area where performance varies by model schema and evaluation method, reinforcing the need for explicit task definitions, benchmarks, and monitoring in regulated workflows (Croxford et al., 2025).

Beyond strong performance on individual NLP tasks, big models enable cross-task reuse, which was previously largely impractical with small, task-specific models. For example, a single LLM can be repurposed through prompting and, when needed, lightweight adaptation to support both long-form summarization and structured information extraction from narrative clinical text (Van Veen et al., 2024; Huang et al., 2024). LLMs can also respond to variable and case-specific reviewer questions. This capability is already being explored in PV settings, such as translating natural-language questions into executable database queries thereby reducing the need to train and maintain multiple models for each scenario. Furthermore, multi-agent frameworks that employ multiple generative AI models, each tailored to specific PV tasks, can be used in combination to enhance analytical capabilities. However, generative AI introduces new challenges, such as hallucinations and unpredictable outputs, due to the inherently probabilistic nature of these models. Mitigating these risks may require additional safeguards, including constrained generation, schema validation, retrieval grounding, uncertainty-aware review processes, and human oversight mechanisms that were less critical in earlier rule-based or narrowly scoped ML systems (Ramcharran et al., 2025).

In contrast, a small model is usually designed for one specific task. For instance, a severity classifier has no built-in capability to summarize narratives, and a deduplication model will not assist with event coding or signal triage. The advantage of this narrow-scope approach is that each small model has a clear purpose, a limited input space, and well-defined outputs, which generally makes it easier to document, validate, and embed within standard operating procedures. Also, small models usually need more task-specific labelled data, but this also means they can be trained on internal historical ICSRs whereas commercial LLMs cannot access or use such data during training due to accessibility. Additionally, the failure modes of small models tend to be more stable and easier to analyze.

Overall, we summarized several common operational tendencies and tradeoffs when considering big or small models under the concepts of computable PV (Table 1). Beyond the different strengths and weaknesses of big and small models, deployment and governance considerations also play critical roles. Large models are computationally more demanding and, when accessed through external APIs, may require careful attention to data protection, jurisdiction, and contractual controls. Running big AI models on every ICSR in a large safety database can also be expensive. In contrast, smaller models are lighter and can often be deployed within existing environments, including on-premises solutions, with clearer boundaries around data flows and responsibilities.

**TABLE 1:** Considerations for big or small models in Computable PV.

Dimension	“Big” models	“Small” models
Technology	LLMs/foundation models	Task-specific ML + rule-based NLP
Task coverage	Can span multiple tasks	Usually single-task components
Adaptation method	Prompting, retrieval augmented generation, light fine-tuning	Supervised training, rule engineering
Outputs	Flexible (free text, structured, or both); can vary with prompts	Constrained, predefined output formats
Reliability/failure modes	Hallucinations, information loss, prompt sensitivity, variable output	Overfitting/underfitting, dataset shift, brittleness to new patterns
Auditability/validation	Harder	Easier
Data requirements	Lower; data access may be restricted to some models (privacy/API constraints)	Higher; can train on internal ICSR data
Cost/compute	Higher	Lower
Best-fit PV tasks	Text-heavy, context-dependent tasks	High-volume, well-defined tasks with stable rules and clear error tolerances
Typical PV use	Summarization, Q&A, cross-step workflow support/orchestration	Extraction, coding, deduplication, completeness checks

For PV, the key question is not whether big or small models are better in general, but which system architecture is appropriate for each task and level of risk. High-volume, well-defined tasks with stable rules and clearly specified error tolerances such as completeness check or identification of likely duplicate pairs often remain best served by smaller, tightly controlled models and deterministic methods that are straightforward to validate and audit. In contrast, text-heavy, context-dependent tasks such as summarizing complex narratives may benefit from large language models. However, the use of LLMs to summarize texts can result in overlooking and missing key relevant information that could adversely affect the interpretation of the safety signal. Increasingly, however, the most practical path is a hybrid, agentic design in which an LLM acts as an orchestrator and natural-language interface, while calling for a set of domain “skills” (tools) that include non-AI functions (document parsing, rule engines, dictionaries/terminologies, workflow logic), retrieval components, and small models for specific tasks. This tool-using pattern helps constrain generative behavior, improves reliability for operations like structured retrieval, and yields clearer audit trails because intermediate tool calls and outputs can be logged and inspected (Painter et al., 2024; FDA, 2025; Xu et al., 2025).

## Computability of common downstream tasks

Having described why PV systems increasingly combine large models, small models, and non-AI “skills,” we now apply the computability lens to common downstream tasks. We treat computability as a spectrum in which some straightforward steps (e.g., completeness checks, deduplication) have clear inputs/outputs and can be audited with deterministic rules or small supervised models whereas other steps such as narrative summarization can benefit from LLMs when constrained by tools and schema validation. For complex and judgment-intensive tasks such as case-level causality assessment, models may help structure evidence, but expert oversight remains essential.

Below we illustrate this with concrete PV workflows and studies.

### Building blocks using small model

Prior AI work in PV has shown that NLP and ML can operationalize many “building-block” tasks. These tasks include extracting clinical features to support case definitions (e.g., anaphylaxis); grouping reports that describe similar conditions using clustering or network-based similarity; extracting time-related details related to drug and adverse event from free text ICSR narratives; and pulling demographic and clinical concepts from narratives (Ball and Dal Pan, 2022). Additional applications include, prioritizing which reports are most informative for causality-focused review (Kreimeyer et al., 2021), detecting potential duplicates, and text mining product labeling to map and encode adverse events for downstream comparisons (Ball and Dal Pan, 2022).

### Information retrieval, extraction and summarization

LLMs have demonstrated strong performance in information retrieval settings, with studies reporting high accuracy in medical literature summarization when properly prompted and validated (Hake et al., 2024). From a computability standpoint, these tasks are attractive because their inputs and outputs can be partially standardized such as retrieving citable source passages, producing constrained summaries, or extracting predefined fields. This enables the evaluation against reference sets and audit trails through logged retrieval results and structured outputs. Studies have also demonstrated that AI systems can extract adverse events, patient demographics, and concomitant medications from ICSR narratives, making automated information extraction a particularly promising application in PV (Kim et al., 2023). In practice, reliability is improved when LLMs are paired with tools such as retrieval, terminology constraints, and schema validation so that outputs are bounded, and discrepancies can be systematically reviewed rather than accepted as free-form generation.

### An illustrative example of case study: small vs. big models for concept extraction from ICSR narratives

Extending this line of work, we recently evaluated both small, transformer-based approaches alongside a “big” model (Llama-4) in a pilot study to identify and extract safety-relevant concepts from ICSR narratives. The study used human-annotated benchmark datasets derived from approximately 800 FAERS reports and an existing VAERS benchmark corpus (Kreimeyer et al., 2022; Foster et al., 2018). For the BioBERT-based named entity recognition (NER) model, 80% of the annotated reports were used for supervised training and the remaining 20% for evaluation. The zero-shot Llama-4 model was evaluated on the same held-out test sets using prompt-based extraction without task-specific fine-tuning. Similar train/test procedures were applied to the VAERS dataset. Performance was evaluated using overall F1-score on boundary-sensitive entity extraction tasks. The full details of this pilot study will be reported separately elsewhere.

Our findings indicate that while general-purpose models show promise, supervised training may offer advantages for the granular and structurally complex requirements of PV tasks. In our evaluation using FAERS narratives, the fine-tuned BioBERT model outperformed the zero-shot Llama-4 LLM in both datasets, achieving a higher overall F1-score of 0.54 compared to 0.42. A similar performance gap was observed in the VAERS vaccine corpus, where BioBERT reached an overall F1 of 0.53 versus 0.31 for the LLM baseline (Table 2). These results are offered as an illustration that task-specific fine-tuning can confer advantages in structured PV extraction tasks, rather than as a definitive comparison of model capability.

**TABLE 2:** Comparing BERT and LLM performance in NER task of extracting safety concepts.

Dataset	Foundation model	Approach	Overall F1-score
FAERS	BioBERT	Supervised fine-tuning	0.54
FAERS	Llama-4	Prompted, zero-shot baseline	0.42
VAERS	BioBERT	Supervised fine-tuning	0.53
VAERS	Llama-4	Prompted, zero-shot baseline	0.31

Error analysis further clarified these failure modes, showing that LLM-derived annotations were prone to hallucinated start and end numbers, boundary drift, and broader label confusion, which complicates their direct use in regulatory workflows where traceability is critical. Conversely, the BERT model acted as a more schema-consistent and conservative “workhorse” that produced more exact matches and fewer spurious predictions. These results support a hybrid model approach within a sociotechnical framework: fine-tuned small models provide stable, high-throughput, and schema-faithful labels for large-scale deployment, while LLMs serve as flexible assistants to bootstrap annotations or support interactive reviewer feedback.

## The frontier of computability: “trust but verify”

The preceding examples show why computable PV is rarely a single model decision. Even when individual subtasks are tractable (rules-based checks, deduplication, extraction, retrieval-augmented summarization), real workflows must still manage residual error, uncertainty, and accountability at decision points that carry clinical and regulatory risk. The frontier is therefore not whether AI can produce an output, but whether that output can be trusted enough to act on, given the available audit trail and validation evidence. This is where a “trust but verify” strategy (Ball et al., 2024) becomes essential: use automation to scale routine, well-specified steps, while requiring structured checks and expert review when the task moves from pattern recognition to judgment.

Standardization through medical coding and deduplication serves as the bedrock of computable PV. Automated systems, such as the ALIGN system, utilize compositional LLM architectures to achieve 87%–90% accuracy in MedDRA coding by employing a multi-step process of candidate generation and self-evaluation (Seedat et al., 2024). Similarly, ICSR deduplication tools leverage probabilistic record linkage of structured and unstructured data to reduce manual workloads by up to 60% (Kreimeyer et al., 2022). In practice, deduplication has seen higher adoption rates because these tools transparently implement algorithms that closely parallel existing human cognitive processes, offering visible relative advantages and high “trialability” for safety reviewers (Ball et al., 2024).

Despite these successes, AI continues to fall short in tasks requiring deep domain expertise, such as case-level causality assessment. Determining whether a drug likely caused a specific adverse event remains a process of expert clinical judgment and global introspection, requiring the integration of nuanced clinical features including temporality, rechallenge, and alternative etiologies against a background of pharmacological mechanisms. Furthermore, the involvement of abstract reasoning along with the interaction of multiple clinical variables depend on the context of the drug and adverse event of interest. Current foundation models often lack the specialized training in regulatory precedents and clinical context to replicate this consistently. This validation gap is exacerbated by subjectivity in human expert classification and a lack of robust, domain-specific benchmarks that measure AI performance against human expert judgment in real-world scenarios. The creation of benchmarks in PV is challenging due to the nuances involved with specific drug-adverse event pairs and the difficulty of generalizing AI models to all possible drug-adverse event combinations. Furthermore, drug-related rare or novel adverse events and syndromes may emerge over time as new drugs enter the market, complicating benchmark development.

A critical barrier to autonomous causality assessment is the fundamental divergence in introspective judgment between machines and humans. In a pilot study applying an LLM to a single well-documented case series, our preliminary findings suggest that AI models can act too confidently, classifying drug-event cases as “causality assessable” in ambiguous scenarios where human reviewers determined they cannot reliably make a causality determination. While there is high agreement on “clear” cases at the extremes, AI is frequently overly optimistic in the “muddled area,” showing a higher propensity to accept cases that humans would reject. This overconfidence is particularly notable given that even human adjudicators only reach full agreement on binary causality classifications in approximately 61% of cases, as mentioned earlier (Han et al., 2017).

The uncertainty associated with model imperfection has direct implications for how much trust we put in the model’s outcome. To manage this, PV systems must distinguish between aleatoric uncertainty (inherent randomness in data) and epistemic uncertainty (limited knowledge), ideally through the construction of confidence intervals (CIs) for model outcomes (Ball et al., 2024). While Explainable AI (XAI) methods such as SHapley Additive exPlanations (SHAP) and Local Interpretable Model-agnostic Explanations (LIME), can improve clinician trust and diagnostic confidence, a survey of clinical decision support systems reveals that these post-hoc explanations often increase cognitive load and may exhibit a misalignment with established domain reasoning processes (Cabitza et al., 2017; Cabitza and Campagner, 2021). Therefore, transparency alone does not guarantee utility; AI outputs must be accurate, contextually relevant and actionable within pharmacovigilance safety evaluators’ workflows.

Importantly, validation in computable PV extends beyond conventional technical performance metrics alone. Technical validation may demonstrate acceptable benchmark accuracy on predefined tasks, but operational deployment in regulated PV workflows also requires clinical and pharmacovigilance expert validation to ensure that outputs preserve medically relevant context, and evidentiary interpretation. For example, an LLM-generated summary may appear linguistically coherent while still omitting clinically important details or overstating causal interpretation in ways that could affect downstream safety assessment. Workflow validation is therefore also necessary to evaluate how AI outputs interact with reviewer decision-making and human oversight processes in operational environments. In addition, governance considerations must determine whether AI-assisted workflows satisfy organizational, regulatory, and accountability requirements for acceptable use in safety surveillance.

Ultimately, a human-centered notion of computable PV treats AI as augmented intelligence rather than a replacement for experts. Consistent with FDA and EMA principles (FDA and EMA, 2026), this requires a sociotechnical framework that connects technology to people and organizational policies. Recent smart-hospital pharmacovigilance frameworks similarly emphasize that effective AI-enabled safety surveillance depends on interoperable data systems, workflow integration, data quality, and expert-guided interpretation rather than model capability alone (De Pretis et al., 2022; De Pretis et al., 2025). In such Human-AI collaborative frameworks, AI handles computational heavy lifting such as real-time deduplication and processing large safety-report volumes, while human experts provide ethical and strategic oversight for complex decisions. Furthermore, AI competence is a prerequisite for informed trust, ensuring that reviewers can critically evaluate AI outputs rather than accepting them uncritically (Ball et al., 2024).

## Future of computable pharmacovigilance

Taken together, our recent experience suggests that the PV cognitive framework is currently partially computable, and that it is not a question about AI, but a property of tasks involved in the PV workflow. Computing the PV cognitive framework does not just involve a series of information processing steps, albeit complex, but must also build an argument convincing to a human expert in a trustworthy manner. In other words, trustworthiness itself is the core of the PV cognitive framework (Nosek et al., 2026), something that, at least for now, only matters to humans, and only humans can judge. Therefore, a more realistic goal today is not to pursue a fully computable PV system, but a human–AI system that remains under human control.

A useful way to interpret “computable PV” is to treat it as a design principle rather than an endpoint. In practice, operationalizing computable PV will likely require phased and risk-based implementation strategies rather than wholesale automation of end-to-end workflows. Workflows should be decomposed into steps with well-established inputs and outputs and then matched to the most appropriate level of automation. Some steps are already good candidates for high confidence computation (e.g., checking minimum case criteria, extracting key safety concepts, and flagging reports that match predefined case definitions). Other activities, most notably the causality assessment, remain difficult to formalize and evaluate in a way that supports consistent and auditable decision-making. Importantly, this does not imply that AI is “not good enough,” but rather that PV systems must explicitly define where uncertainty and value judgments enter, and how they will be governed (Agrawal et al., 2019). Such staged deployment approaches allow organizations to incrementally evaluate model reliability, define thresholds for human intervention, and refine governance frameworks as both model capabilities and regulatory expectations continue to evolve.

This perspective also reframes the “big versus small model” distinction. The terms are convenient shorthand for capabilities and validation properties, but real PV systems increasingly use orchestrated, risk-based architectures in which performance depends not only on model size, but also on task definition, annotation quality, prompting or fine-tuning strategy, integrating and grounding with relevant knowledge base, and evaluation methodology. In practice, large models often act as flexible interfaces for narrative understanding and reasoning, while smaller task models, rule engines, terminologies, retrieval components, and schema validators provide bounded operations with clearer audit trails and more predictable behavior. Emerging agentic AI frameworks further extend this paradigm by enabling large models to dynamically select and coordinate specialized tools, including smaller models and non-AI functions, depending on the context and task requirements. Rather than representing competing paradigms with fixed advantages, “big” and “small” models are increasingly complementary components within hybrid human–AI PV systems. From a computability standpoint, such decomposition is beneficial because it makes intermediate steps explicit and evaluable, rather than burying the entire process inside unconstrained generation.

At the same time, the relevant technical frontier is moving quickly. Foundation models are increasingly paired with domain knowledge add-ons (e.g., curated reference libraries, retrieval over internal corpora, structured ontologies, and case-based memory) and agent-like orchestration patterns that select tools, plan multi-step analyses, and self-check intermediate outputs. These developments may expand the portion of PV that can be treated as reliably computable not necessarily by “solving” causality in a single leap, but by improving the system’s ability to assemble evidence, surface alternative explanations, and quantify uncertainty in ways that are meaningful to expert review. As a result, parts of this perspective are inherently time-bounded in that the boundary between computable assistance and autonomous action may shift as model capabilities, evaluation methods, and governance standards mature.

In that sense, computable PV should not be framed as an “end-of-history” claim in which PV approaches full automation. It is better understood as a pragmatic roadmap that describes which tasks can be computed with acceptable risk, specifying how outputs will be evaluated and monitored, choosing architectures that balance flexibility with auditability, and embed AI within sociotechnical systems that preserve accountability. We may indeed be approaching the end of purely manual, labor-intensive ICSR processing where generative AI is already enabling substantial automation of routine, well-specified steps that have historically dominated PV workflows. However, rather than an “end of history,” this is better viewed as a shift in what PV practice can achieve where human expertise becomes more strategic and oversight-focused, while AI increasingly supports the computational heavy lifting. The future lies in replacing AI-averse workflows with AI-enabled workflows that remain accountable to human experts.

## Data Availability

The original contributions presented in the study are included in the article/supplementary material, further inquiries can be directed to the corresponding author.
